# Supplementary material for: Colony specificity and starvation-driven changes in activity patterns of the red ant Myrmica rubra
Source: PLoS One. 2022 Aug 12;17(8):e0273087. doi: 10.1371/journal.pone.0273087 (PMC9374231; doi:10.1371/journal.pone.0273087)
Supplement: S1 Table — For each colony, tests are performed using all the daily activity indices measured during the satiation phase. P-values in bold are <0.05. (DOCX) [file pone.0273087.s001.docx]

**S1 Table. Spearman correlation tests** between the activity measured at 9am in one zone (nest or foraging area) and the number of ants in the corresponding zone at 9am. For each colony, tests are performed using all the daily activity indices measured during the satiation phase. P-values in bold are <0.05.
