# Supplementary material for: Colony specificity and starvation-driven changes in activity patterns of the red ant Myrmica rubra
Source: PLoS One. 2022 Aug 12;17(8):e0273087. doi: 10.1371/journal.pone.0273087 (PMC9374231; doi:10.1371/journal.pone.0273087)
Supplement: S2 Table — Spearman tests were not performed for the recovery phase due to the too few daily activity indices available per colony. P-values in bold are <0.05. (DOCX) [file pone.0273087.s002.docx]

**S2 Table. Spearman correlation tests** between the daily activity indices measured inside and outside of the nest for each colony during the satiation phase (n=4 per colony) and the starvation phase (n=6 per colony). Spearman tests were not performed for the recovery phase due to the too few daily activity indices available per colony. P-values in bold are <0.05.
