# Supplementary material for: Colony specificity and starvation-driven changes in activity patterns of the red ant Myrmica rubra
Source: PLoS One. 2022 Aug 12;17(8):e0273087. doi: 10.1371/journal.pone.0273087 (PMC9374231; doi:10.1371/journal.pone.0273087)
Supplement: S3 Table — diff = difference in average value between the two phases. Phase 1 = Satiation phase, Phase 2 = Starvation phase and Phase 3 = Recovery phase. CI = Confidence Interval. P-values in bold are <0.05. (DOCX) [file pone.0273087.s003.docx]

**S3 Table. Results of the Tukey HSD test** performed on the activity inside the nest, the activity in the foraging area, the coefficients of variation and the number of peaks measured in every pair of experimental phases. diff = difference in average value between the two phases. Phase 1 = Satiation phase, Phase 2 = Starvation phase and Phase 3 = Recovery phase. CI = Confidence Interval. P-values in bold are <0.05.
