# Supplementary material for: Colony specificity and starvation-driven changes in activity patterns of the red ant Myrmica rubra
Source: PLoS One. 2022 Aug 12;17(8):e0273087. doi: 10.1371/journal.pone.0273087 (PMC9374231; doi:10.1371/journal.pone.0273087)
Supplement: S5 Table — Pairwise t-test comparisons with corrected p-values (Bonferroni method). P-values in bold are <0.05. (DOCX) [file pone.0273087.s005.docx]

**S5 Table. Daily comparisons of the activity in the foraging area for the satiation phase and the starvation phase separately.** Pairwise t-test comparisons with corrected p-values (Bonferroni method). P-values in bold are <0.05.
